# Clinical and Pathological Features and Gene Expression Profiles of Clinically Aggressive Papillary Thyroid Carcinomas

**DOI:** 10.1007/s12022-023-09769-x

**Published:** 2023-05-19

**Authors:** Jasna Metovic, Francesco Cabutti, Simona Osella-Abate, Giulia Orlando, Cristian Tampieri, Francesca Napoli, Francesca Maletta, Lorenzo Daniele, Marco Volante, Mauro Papotti

**Affiliations:** 1 Department of Oncology, University of Turin, Città Della Salute E Della Scienza Hospital, Pathology Unit, Turin, Italy; 2 Department of Medical Sciences, University of Turin, Città Della Salute E Della Scienza Hospital, Pathology Unit, Turin, Italy; 3 Pathology Unit, Città della Salute e della Scienza Hospital, Turin, Italy; 4 Department of Oncology, University of Turin, San Luigi Hospital, Orbassano, Turin, Italy; 5 Pathology Unit, Mauriziano Hospital, Turin, Italy

**Keywords:** Papillary thyroid carcinoma, Prognosis, Gene expression profiling, Nanostring

## Abstract

Papillary thyroid carcinoma (PTC) is considered an indolent neoplasm but it may demonstrate aggressive behavior. We aimed to identify clinical and pathological characteristics and molecular signatures associated with aggressive forms of PTCs. We selected 43 aggressive PTC cases based on the presence of metastases at the time of diagnosis, the development of distant metastasis during follow-up, and/or biochemical recurrence, and 43 PTC patients that were disease-free upon follow-up, matching them according to age, sex, pT, and pN parameters. Twenty-four pairs (a total of 48 cases) and 6 normal thyroid tissues were studied using targeted mRNA screening of cancer-associated genes employing NanoString nCounter^®^ technology. In general, aggressive PTCs showed distinctive clinical and morphological features. Among adverse prognostic parameters, the presence of necrosis and an increased mitotic index were associated with shorter disease-free and overall survivals. Other parameters associated with shorter disease-free or overall survivals include a lack of tumor capsule, the presence of vascular invasion, tumor-infiltrating lymphocytes, fibrosclerotic changes, age > 55 years, and a high pTN stage. Various pathways were differentially regulated in non-aggressive as compared to aggressive PTC, including the DNA damage repair, the MAPK, and the RAS pathways. In particular, the hedgehog pathway was differentially de-regulated in aggressive PTC as compared to non-aggressive PTC cases, being *WNT10A* and *GLI3* genes significantly up- and down-regulated in aggressive PTC and *GSK3B* up-regulated in non-aggressive PTC cases. In conclusion, our study revealed specific molecular signatures and morphological features in aggressive PTC that may be useful to predict more aggressive behavior in a subset of PTC patients. These findings may be useful when developing novel, tailored treatment options for these patients.

**Supplementary Information:**

The online version contains supplementary material available at 10.1007/s12022-023-09769-x.

## Introduction

Papillary thyroid carcinoma (PTC) derives from follicular cells and is the most common thyroid malignancy, comprising more than 80% of all malignant thyroid tumors [1–3]. PTC is generally considered a tumor with indolent behavior, slow progression, and low mortality rates [4]. However, a subset of cases may present an aggressive clinical course. Clinical and morphological features and gene alterations allow us to predict the aggressive behavior of this tumor, but only up to a certain extent [5]. Compared to classical PTCs, the hobnail, tall cell, columnar, and solid subtypes predominate in aggressive cases [6, 7]. Since several years, it has been ascertained that prognosis is worsened by higher disease stages, tumor size above 3–4 cm, presence of distant metastases, extrathyroidal extension, neoplastic infiltration of the resection margins, and/or older age at diagnosis [8, 9].

In terms of the impact of the molecular signature on prognosis, various studies have attempted to better characterize the genetic make-up of these neoplasms. The *BRAF* p.V600E mutation is the most common genomic alteration in PTC (found in approximately 70% of cases). It is currently considered that the *BRAF* mutation by itself is not a high-risk factor, but it has a strong adverse prognostic impact in association with *TERT* promoter mutations [10, 11]. Mutations of *NRAS*, *KRAS*, and *HRAS* are also common [12], especially in the follicular variant [13], but lack a significant and independent prognostic role. Other molecular alterations accumulate in PTC during cancer progression, such as those in *TP53*, *PIK3CA*, and *AKT1* [14, 15]. In this context, *TERT* promoter mutations (C228T and C250T) are present in more advanced PTCs and are strongly associated with a higher risk of distant metastases, recurrence, and cancer-related mortality [10, 16].

Apart from genomic alterations, some studies investigated gene or microRNA expression profiling in PTC. However, only a few studies were designed with the specific purpose of characterizing PTCs with aggressive clinical features. The most recent data are obtained by analysis of publicly available datasets (i.e., TCGA) but are affected by the lack of a detailed characterization of cases and by an unbalanced selection of cases with or without aggressive features [17–22].

**Fig. 1:**
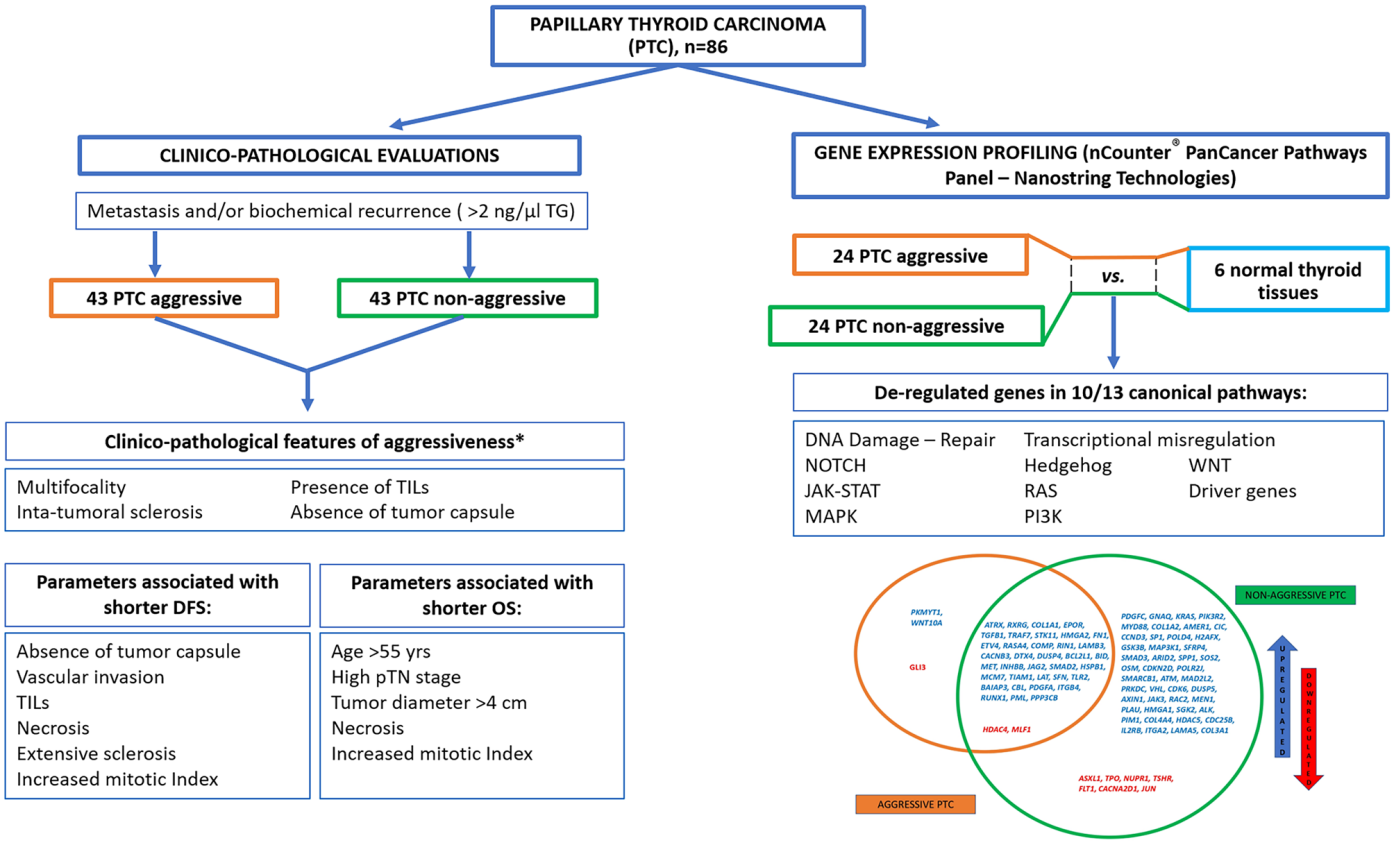
Summary of design and main results of the study

We therefore aimed to test the profiles of gene expression through the NanoString nCounter^®^ technology in a series of well-characterized clinically aggressive PTC, matched with non-aggressive PTC cases, to identify molecular biomarkers potentially predictive of an aggressive clinical course that might be integrated with the current pathological characterization. A summary of the study design and main results is illustrated in Fig. 1.

## Material and Methods

### Case Selection

A group of 43 cases with aggressive clinical behavior, defined based on the presence of metastasis at diagnosis, development of distant metastases during follow-up, and/or biochemical recurrence (> 2 ng/ml levels of thyroglobulin after TSH stimulation), was selected from a large series of PTC undergoing thyroidectomy from 2002 to 2017 at “Città della Salute e della Scienza” Hospital (Turin, Italy), San Luigi Gonzaga Hospital (Orbassano, Turin, Italy), and Mauriziano Hospital (Turin, Italy). Six cases had a biochemical recurrence, only. Thirty-seven cases had metachronous metastases. These latter cases included one case with synchronous lung metastases, only, four cases with metachronous multiorgan dissemination (including lymph nodes lung, brain, bone), and 32 cases with metachronous lymph node involvement, with or without infiltration of soft tissues in the cervical neck. These cases were matched for age, sex, and T and N parameters with 43 controls, undergoing surgery in the same period, which resulted in free of disease during the clinical follow-up (median follow-up 12.1 years, range 4.4–19 years). Characteristics of lymph node metastases in both aggressive and non-aggressive cases are reported in Supplementary Table 1.

**Fig. 2:**
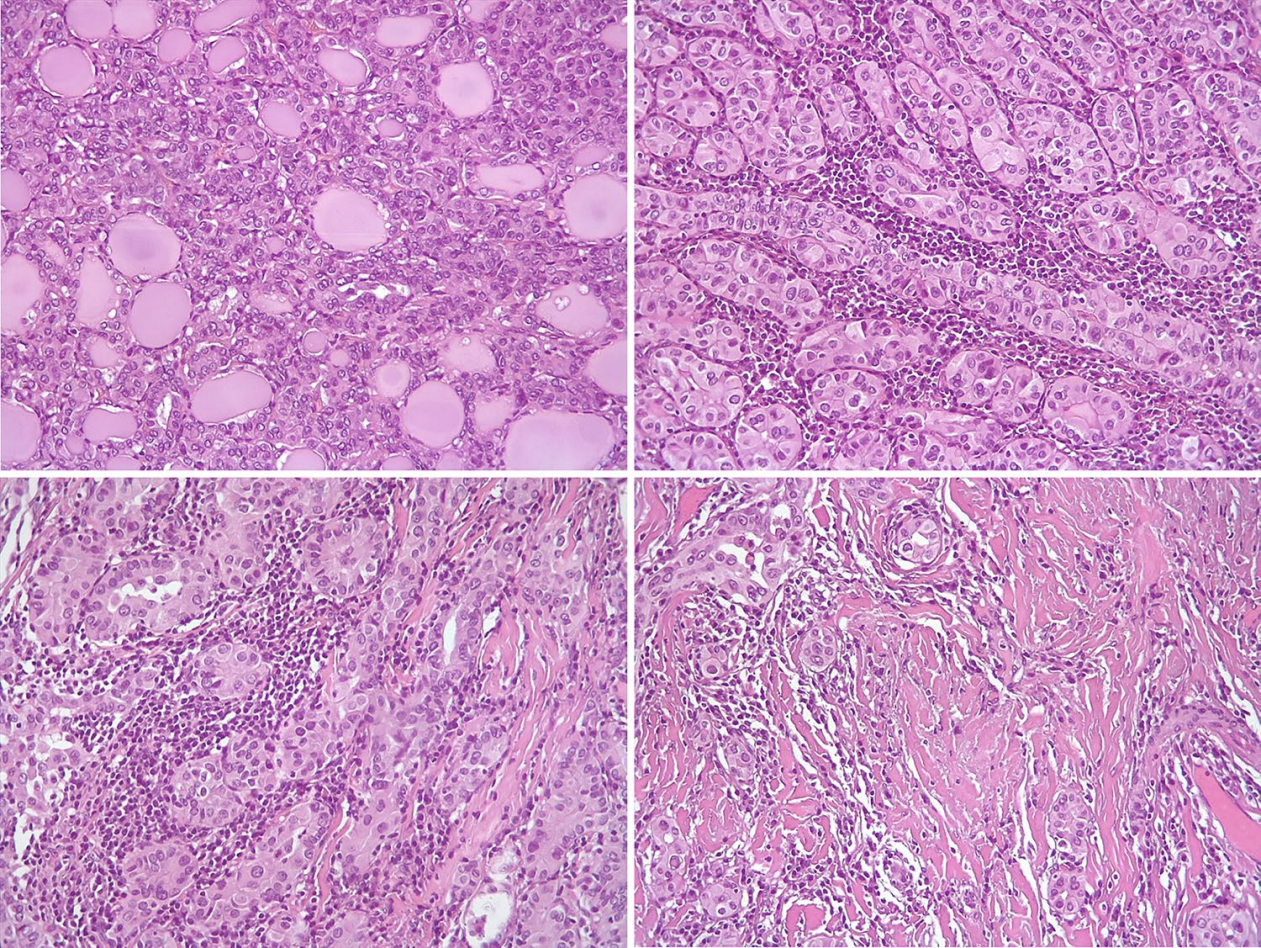
Representative images of the distribution of tumor-infiltrating lymphocytes and fibro-sclerotic changes in papillary thyroid carcinoma cases. Top left: tumor-infiltrating lymphocytes and fibro-sclerotic changes absent; top right: tumor-infiltrating lymphocytes present, brisk, and fibro-sclerotic changes absent; bottom left: tumor-infiltrating lymphocytes present, brisk, and fibro-sclerotic changes present mild; bottom right: tumor-infiltrating lymphocytes present, non-brisk, and fibro-sclerotic changes present extensively

Representative hematoxylin and eosin (H&E) stained slides were re-evaluated in all cases enrolled in the study by three of us (FM, MV, MP). The following parameters were re-assessed and recorded: histological subtype, TNM stage according to AJCC 8th edition, tumor diameter, uni- or multifocal presentation (including bilaterality), presence of tumor capsule, presence and extent of microscopic extrathyroidal invasion, presence of angioinvasion, status of surgical margins, presence of necrosis, presence of tumor-infiltrating lymphocytes (TILs), and presence of fibro-sclerotic changes. TILs were coded as absent, present “non-brisk,” and present “brisk” according to standard evaluation methods applied in breast cancer [23]. For statistical purposes, cases were grouped as TILs absent or present, in this latter case including both non-brisk and brisk patterns. Fibro-sclerotic changes were coded as mild (in the presence of thin and rare bands of collagen deposition) or extensive (in the presence of more abundant and thick fibrous bands). Representative images of the different patterns of distribution of TILs and fibro-sclerotic changes are illustrated in Fig. 2. Mitotic count was determined as the number of mitotic figures in 2 mm^2^. Five cases (four in the aggressive PTC group and one in the non-aggressive PTC group) met the criteria for high-grade differentiated thyroid carcinomas because of the presence of coagulative necrosis. One case had also a mitotic index of 5 in 2 mm^2^. However, due to their limited number, these cases were not further considered as a separate group but necrosis and mitotic index were considered individually for the sake of statistical analyses.

A pool of thyroid tissues devoid of macroscopically recognizable alterations from patients operated on due to follicular nodular disease was used as the baseline for gene expression testing and consisted of 3 female (aged 33, 50, and 71) and 3 male patients (aged 40, 50, and 69).

Before the study started, all cases were de-identified and coded by a pathology staff member not involved in the study, and all data were accessed anonymously. The study was approved by the local Ethical Committee (#610, date December 20th, 2017) and conducted in accordance with the principles set out in the Declaration of Helsinki. Considering the retrospective nature of this research protocol and that it had no impact on patients’ care, no specific written informed consent was required.

### Molecular Analyses

For molecular testing, 24 pairs of aggressive and their non-aggressive match were analyzed by means of gene expression profiling (for a total of 48 cases tested) along with the pool of 6 samples of non-neoplastic thyroid tissue. In this sub-series, the aggressive cases were also matched with non-aggressive cases for the histological subtype.

For RNA extraction, two 10-μm-thick formalin-fixed and paraffin-embedded (FFPE) tissue sections were obtained from each tissue block and collected in sterile Eppendorf tubes by means of manual microdissection. The same procedure was adopted for normal thyroid tissues, making sure that only healthy/unaltered parenchymal areas were dissected. RNA isolation was performed using the FFPE RNA Isolation Kit (Roche Diagnostics GmbH, Mannheim, Germany), according to the manufacturer’s protocols. Total RNA concentration was assessed using a NanoDrop spectrophotometer (Thermo Fisher Scientific, Inc., Wilmington, DE, USA).

NanoString nCounter^®^ technology was used to detail the transcriptomic cancer-associated landscape in PTC. Three hundred nanograms of total RNA from each sample were hybridized to the nCounter PanCancer Pathways Panel, according to the manufacturer’s instructions (NanoString Technologies, Seattle, WA, USA). The nCounter PanCancer Pathways panel includes 730 genes from 13 canonical pathways and 40 selected reference genes. The analyses were set up according to the protocol provided by the manufacturer. Expression data were normalized and analyzed with the nSolver Analysis Software (version 4.0.62). For background correction, the mean count of negative controls plus two times the standard deviation was subtracted from the counts of each gene. The means of the supplied positive controls and the geometric mean of the housekeeping genes were used to normalize the measured expression values. Both positive and negative controls were included in the panel, according to the manufacturer’s instructions. Additionally, the advanced analysis module (version 2.0.134) was used to perform differential expression analyses. Briefly, by the differential expression analysis tool (nSolver Advanced Analysis module), for each gene, a single linear regression was fit using all selected covariates to predict expression. Volcano plots were generated to display each gene’s -log10 (*p*-value) and log2 fold change with the selected covariate. Highly statistically significant genes fell at the top of the plot above the horizontal lines, and highly differentially expressed genes fell to either side. Horizontal lines indicated various *p*-value thresholds.

### Statistical Analyses

All analyses were performed using Stata/MP 15.0 Statistical Software (STATA, College Station, TX). The differences in the distribution of clinical-pathological variables were analyzed using parametric and non-parametric tests (student’s t test, Pearson’s chi-square test, Bonferroni’s correction, Wilcoxon’s rank test). Overall survival (OS) was calculated from the surgical excision date of the primary tumor to the date of death or last check-up. The disease-free survival (DFS) was calculated from the date of surgical excision of the primary tumor to the date of the first relapse or last check-up. Survival curves were plotted using the Kaplan-Meier method, and the statistical comparisons were performed using the log-rank test. The Cox regression analyses were carried out on DFS and OS to calculate HRs and 95% CIs for the different study groups. Cox proportional hazards regression model was used in multivariate survival analysis to assess the independent role of parameters significant at univariate analysis. All statistical tests were two sided. A *p*-value < 0.05 was considered significant.

## Results

### Clinical and Pathological Characteristics

As shown in Table 1, aggressive PTC was more frequently multifocal (26/43, 60.5%) as compared to non-aggressive cases (16/43, 37.2%) (*p* = 0.031). The tumor capsule was absent in more than half of aggressive cases (29/43, 67.4%) and in only 17/43 (39.5%) of non-aggressive ones (*p* = 0.015). Furthermore, aggressive PTCs had more frequent TILs (26/43, 60.5%) as compared to the non-aggressive group (11/43, 25.6%) (*p* = 0.001). Only 10/43 (23.3%) non-aggressive PTCs had extensive fibro-sclerotic changes, as compared to 21/43 (48.8%) aggressive cases (*p* = 0.047). Although not statistically significant, angioinvasion was observed more commonly in aggressive (30/43, 69.8%) as compared to non-aggressive cases (21/43, 48.8%) (*p* = 0.077). Of note, at follow-up, 11/43 (25.6%) patients with aggressive PTC died (8 of their PTC and 3 due to other causes), as compared to 4/43 (9.3%) patients with non-aggressive PTC who died of other causes, all not related to their PTC (*p* = 0.047).

**Table 1:** Clinico-pathological features of the papillary carcinoma case series investigated

**Parameter**		**Total**	**Non-aggressive PTC**	**Aggressive PTC**	***P***-**value**
**Age**	Median (interval)	43 (18–82)	40 (18–82)	46 (19–78)	/
**Sex**	M	32	16	16	/
	F	54	27	27	
**Type of surgery**	Lobectomy	3	2	1^c^	> 0.99
	Total thyroidectomy	83	41	42	
**RAI treatment after surgery (20 missing)**	no	5	5	0	0.15
	yes	61	35	26	
**T**	1	42	21	21	/
	2	22	11	11	
	3	18	9	9	
	4	4	2	2	
**N**	0	34	17	17	/
	1	52	26	26	
**Subtype**	Classic	47	23	24	0.472
	Follicular^a^	14	8	6	
	Oncocytic	5	3	2	
	Tall cell	5	1	4	
	Hobnail	7	2	5	
	Solid	4	3	1	
	Cystic	2	2	0	
	Diffuse sclerosing	2	1	1	
**Subtype (dichotomized)**	Classic/follicular^a^	61	31	30	0.812
	Other subtypes	25	12	13	
**Tumor diameter (cm)**	≤ 1	17	10	7	0.322
	> 1– ≤ 2	37	16	21	
	> 2– ≤ 4	25	15	10	
	> 4	7	2	5	
**Multifocal presentation**	No	44	27	17	0.031
	Yes	42	16	26	
**Bilateral presentation**	No	57	32	25	0.110
	Yes	29	11	18	
**Tumor capsule**	Absent	46	17	29	0.015
	Incomplete	29	17	12	
	Complete	11	9	2	
**Extrathyroidal extension**	No	38	22	16	0.193
	Yes	48	21	27	
**Angioinvasion**	No	34	21	13	0.077
	Yes	52	22	30	
**Surgical margins**	Negative	61	33	28	0.235
	Positive	25	10	15	
**Necrosis**	no	81	42	39	0.167
	yes	5	1	4	
**Mitosis**	0	8	6	2	0.255
	1	72	36	36	
	2	3	1	2	
	3	2	0	2	
	5	1	0	1	
**Tumor infiltrating lymhocytes**	Absent	49	32	17	0.001
	Present^b^	37	11	26	
**Fibro-sclerotic changes**	Absent	24	13	9	0.047
	Mild	33	20	13	
	Extensive	31	10	21	
**Follow-up status**	Alive	71	39	32	0.047
	Dead	15	4	11	

*PTC* papillary thyroid carcinoma, *F* female, *M* male, *RAI* radio-iodine treatment

^a^Including both infiltrative follicular variant and invasive encapsulated follicular variant of papillary carcinoma

^b^Including both “non-brisk” and “brisk” patterns

^c^Subsequent completion lobectomy

**Fig. 3:**
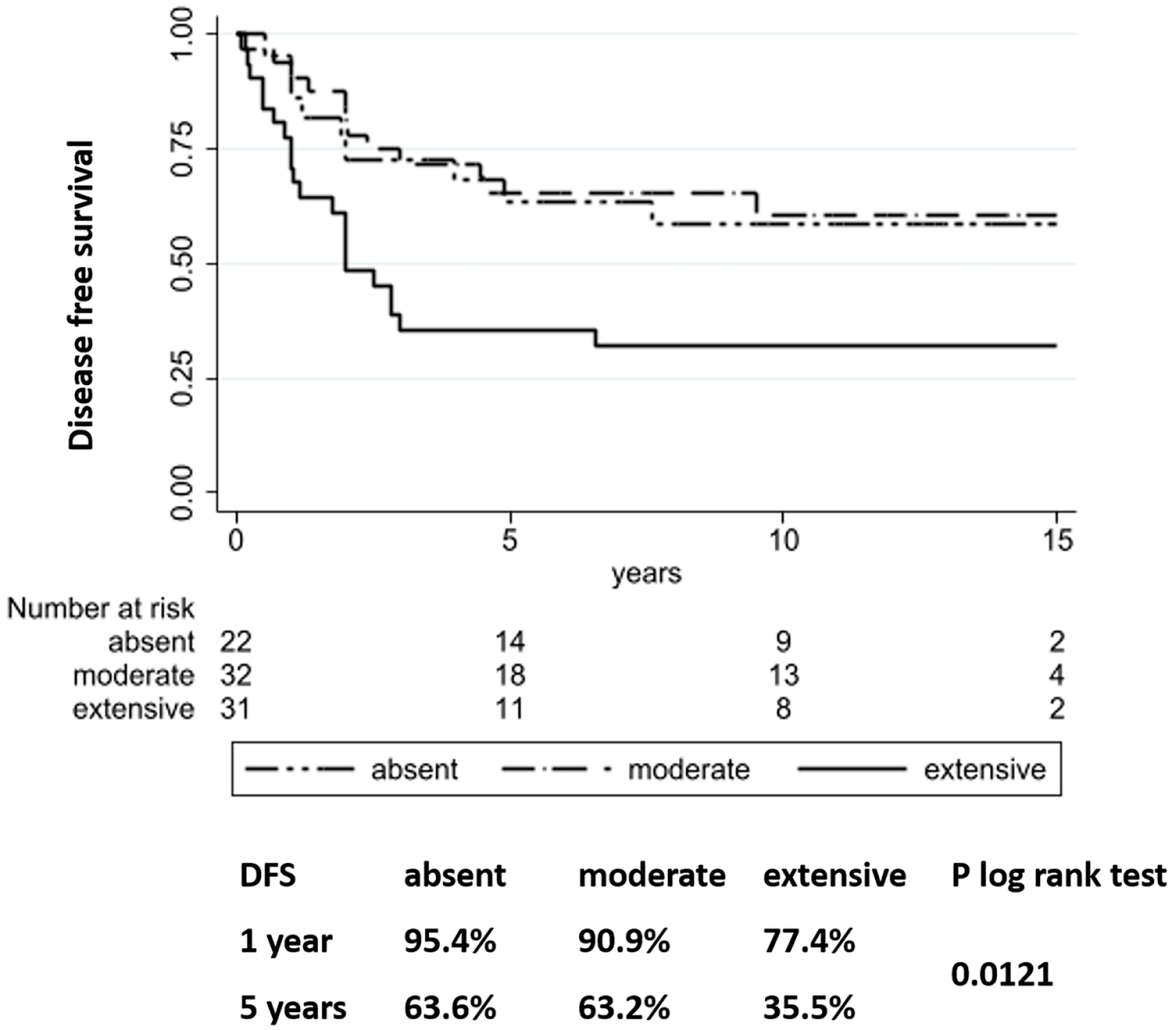
Kaplan-Meier estimates of disease-free survival (DFS) according to the fibro-sclerotic intratumoral changes (coded as absent, mild or extensive)

By means of Cox univariate analyses (Table 2), in terms of DFS, the presence of angioinvasion (*p* = 0.019), TILs (*p* = 0.001), necrosis (*p* = 0.050), and higher mitotic counts (*p* = 0.001) were all correlated with an adverse prognosis. Extensive fibro-sclerotic changes were also associated with shorter DFS (*p* = 0.038 and *p* = 0.021 in the log rank test, Fig. 3). On the other hand, the presence of a complete tumor capsule was a favorable morphologic feature (*p* = 0.029). In terms of OS, tumor diameter larger than 4 cm (*p* = 0.004), presence of necrosis (*p* < 0.001), and higher mitotic index (*p* = 0.001) were parameters correlated with adverse prognosis in terms of OS. However, none of the parameters significant in univariate analyses showed independent statistical significance in multivariate analyses.

**Table 2:** Univariate analysis of disease-free and overall survival in the complete series of non-aggressive and aggressive PTC

		**DFS**			**OS**		
	**Parameter**	**HR**	**CI**	***P* value**	**HR**	**CI**	***P* value**
**Tumor diameter (cm)**	≤ 1	1			1		
	1–2	1.43	0.60–3.39	0.412	0.44	0.06–3.10	0.407
	> 2–4	0.91	0.35–2.38	0.844	2.56	0.51–12.7	0.251
	> 4	2.55	0.81–8.04	0.111	11.1	2.11–58.5	0.004
**Multifocal presentation**	Multi vs uni	1.84	0.99–3.43	0.051	0.74	0.26–2.09	0.571
**Bilateral presentation**	Yes vs no	1.44	0.78–2.66	0.245	1.05	0.36–3.10	0.922
**Tumor capsule**	Absent	1			1		
	Incomplete	0.52	0.26–1.03	0.061	0.99	0.32–3.04	0.986
	Complete	0.20	0.05–0.85	0.029	1.24	0.26–5.84	0.789
**Extrathyroidal extension**	Yes vs no	1.66	0.89–3.10	0.110	0.77	0.28–2.12	0.614
**Angioinvasion**	Yes vs no	1.99	1.12–3.55	0.019	2.47	0.95–6.45	0.064
**Tumor infiltrating lymhocytes**	Present* vs absent	2.81	1.50–5.27	0.001	0.30	0.08–1.06	0.061
**Surgical margins**	Positive vs negative	1.74	0.92–3.27	0.085	1.40	0.48–4.11	0.537
**Necrosis**	Yes vs no	2.81	0.99–7.93	0.050	12.9	3.85–43.6	< 0.001
**Fibro-sclerotic changes**	Absent	1			1		
	Mild	0.91	0.38–2.15	0.828	1.44	0.36–5.77	0.604
	Extensive	2.29	1.05–5.02	0.038	1.46	0.36–5.86	0.589
**Mitotic index**	Linear	2.06	1.37–3.10	0.001	2.29	1.54–3.41	0.001

*DFS* disease-free survival, *OS* overall survival, *HR* hazard ratio, *CI* confidential intervals

*Including both “non-brisk” and “brisk” patterns

**Fig. 4:**
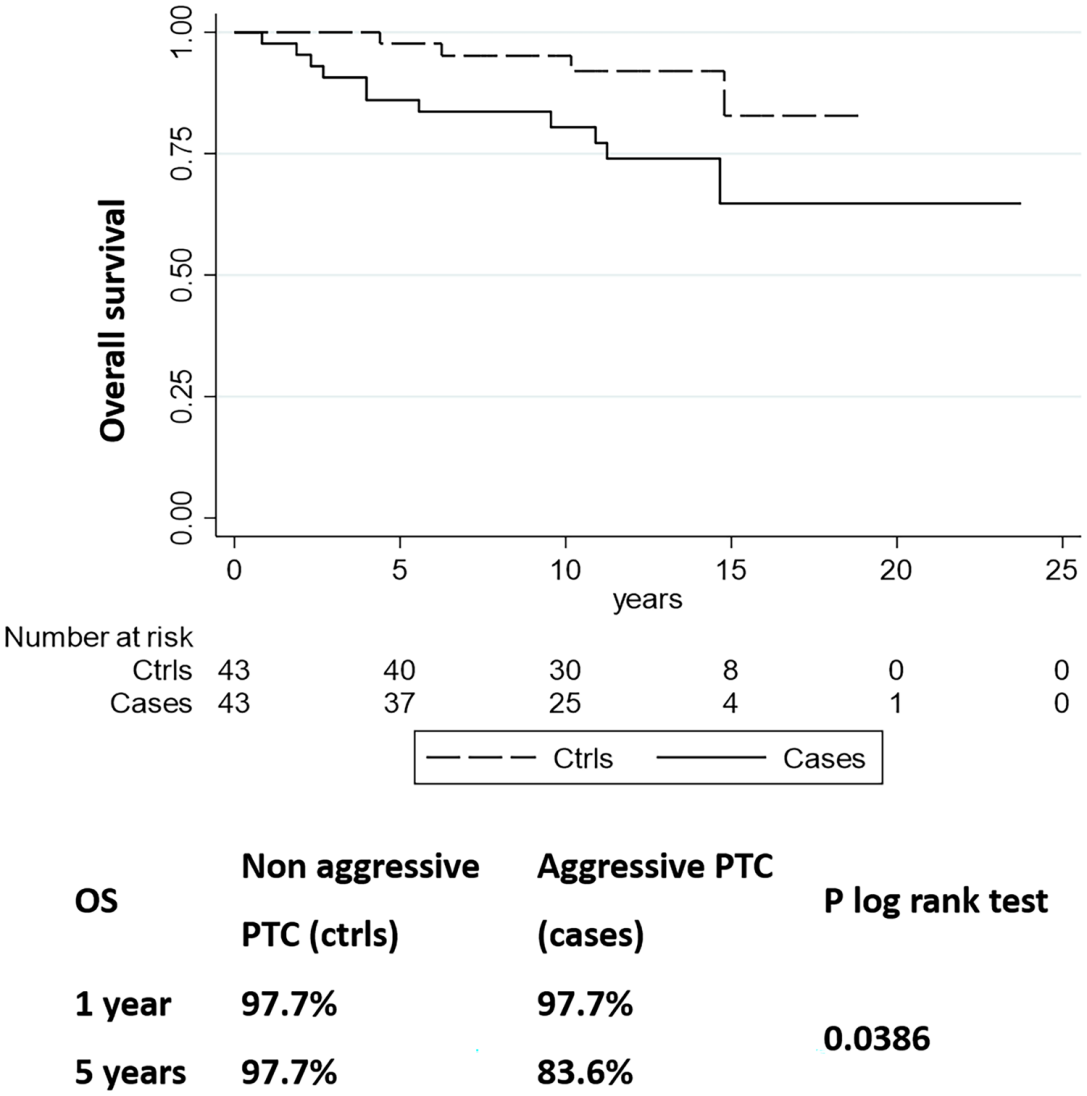
Kaplan-Maier estimates of overall survival (OS) of 43 aggressive versus 43 non-aggressive papillary thyroid carcinoma cases

OS in patients with aggressive PTC was significantly shorter than in patients with non-aggressive PTC (*p* = 0.038) (Fig. 4).

### Gene Expression Profile of Aggressive and Non-Aggressive PTC Cases

The clinical and pathological characteristics of the 48 patients analyzed for gene expression profiling are summarized in Supplementary Table 2.

**Fig. 5:**
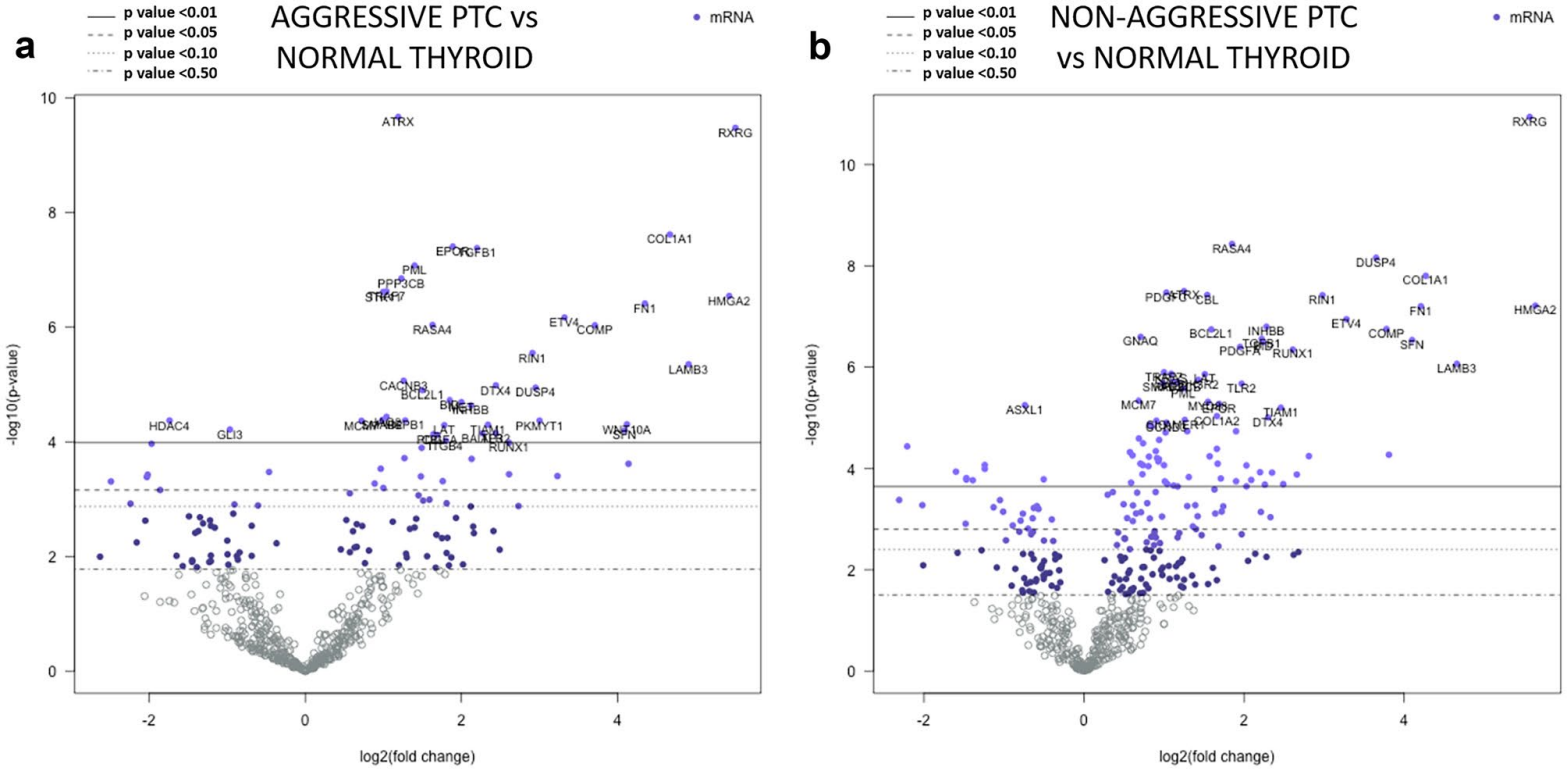
Volcano plot representing differential gene expression profile in **a** aggressive papillary thyroid carcinomas versus normal thyroid tissue and **b** non-aggressive papillary thyroid carcinoma versus normal thyroid tissue

**Fig. 6:**
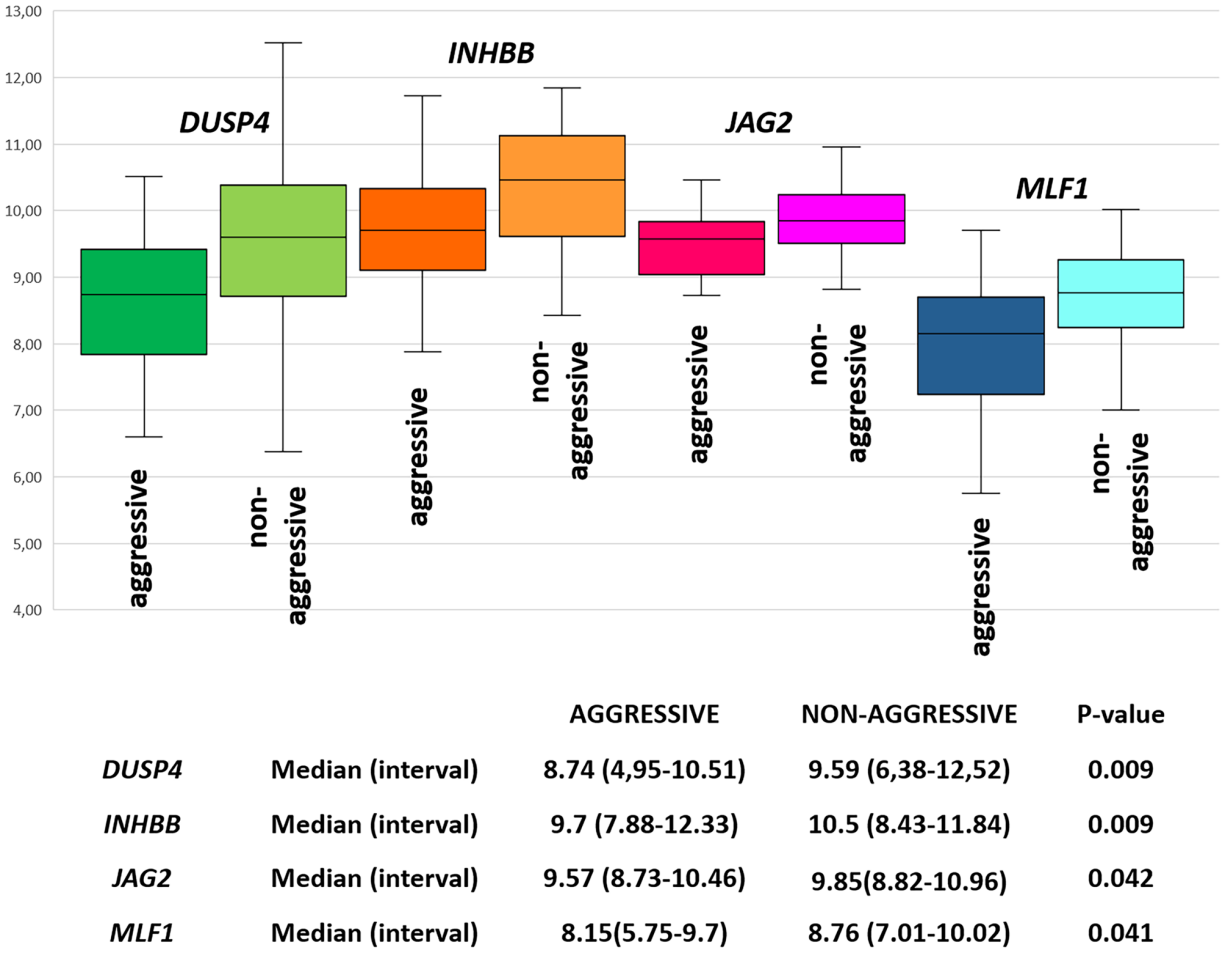
Differential expression of *DUSP4*, *INHBB*, *JAG2*, *MLF1*, in aggressive versus non-aggressive papillary thyroid carcinomas

In aggressive PTC, 38 genes were significantly upregulated and 3 were significantly downregulated as compared to the non-neoplastic tissue (Supplementary Table 3; volcano plot image in Fig. 5a). In non-aggressive PTC, 80 genes were significantly upregulated and 9 were significantly downregulated as compared to non-neoplastic thyroid tissue (Supplementary Table 4; volcano plot image in Fig. 5b). Three genes, only, were significantly de-regulated in aggressive but not in non-aggressive PTC, namely two upregulated (*PKMYT1* and *WNT10A*) and one downregulated (*GLI3*) (Table 3). By contrast, 45 genes were significantly upregulated and seven were downregulated in non-aggressive, but not in aggressive PTC. Thirty-six upregulated and two downregulated genes were in common between aggressive and non-aggressive PTC cases. However, even in this subset, four genes (*DUSP4*, *INHBB*, *JAG2*, and *MLF1*) were significantly upregulated in the group of non-aggressive cases as compared to the aggressive ones (Fig. 6).

**Table 3:** Distribution of deregulated genes, as compared to normal thyroid tissue, in aggressive and non-aggressive PTC cases

	**Up-regulated genes**	**Down-regulated genes**
**Aggressive PTC**	PKMYT1, WNT10A	GLI3
**Both groups**	ATRX, RXRG, COL1A1, EPOR, TGFB1, TRAF7, STK11, HMGA2, FN1, ETV4, RASA4, COMP, RIN1, LAMB3, CACNB3, DTX4, DUSP4, BCL2L1, BID, MET, INHBB, JAG2, SMAD2, HSPB1, MCM7, TIAM1, LAT, SFN, TLR2, BAIAP3, CBL, PDGFA, ITGB4, RUNX1, PML, PPP3CB	HDAC4, MLF1
**Non-aggressive PTC**	PDGFC, GNAQ, KRAS, PIK3R2, MYD88, COL1A2, AMER1, CIC, CCND3, SP1, POLD4, H2AFX, GSK3B, MAP3K1, SFRP4, SMAD3, ARID2, SPP1, SOS2, OSM, CDKN2D, POLR2J, SMARCB1, ATM, MAD2L2, PRKDC, VHL, CDK6, DUSP5, AXIN1, JAK3, RAC2, MEN1, PLAU, HMGA1, SGK2, ALK, PIM1, COL4A4, HDAC5, CDC25B, IL2RB, ITGA2, LAMA5, COL3A1	ASXL1, TPO, NUPR1, TSHR, FLT1, CACNA2D1, JUN

The differential gene expression between aggressive and non-aggressive cases according to different pathways is presented in Supplementary Fig. 1. None of the genes related to DNA damage repair (*POLD4, H2AFX, POLR2J, ATM, MAD2L2, PRKDC*) that were upregulated in non-aggressive PTC cases were found differentially expressed in aggressive PTC (Supplementary Fig. 1a1/2).

Two genes related to the NOTCH pathway, *DTX2* and *JAG2*, were upregulated (Supplementary Fig. 1b1/2) in both groups. All genes de-regulated in the aggressive PTC group and belonging to the transcriptional misregulation pathway were also de-regulated in the non-aggressive PTC group (Supplementary Fig. 1c1/2).

Deregulation of the hedgehog pathway involved different genes in aggressive and non-aggressive PTC. In fact, *WNT10A* and *GLI3* were found up- and downregulated in aggressive PTCs, whereas *GSK3B* was found upregulated in non-aggressive PTCs (Supplementary Fig. 1d1/2).

The analyses of the WNT pathway revealed overlap in only one gene between the two groups (*PPP3CB*), whereas several other genes were found to be de-regulated in non-aggressive cases (*CCND3, GSK3B, SFRP4, SMAD3, AXIN1, RAC2*, *JUN)* (Supplementary Fig. 1e1/2).

Regarding the JAK-STAT pathway, upregulation of *EPOR*, *BCL2L1*, and *CBL* genes was found upregulated in both groups, while *PIK3R2*, *CCND3*, *SOS2*, *OSM*, *JAK3*, *PIM1*, and *IL2RB* genes were up- and *TPO* down-regulated in the non-aggressive group, only (Supplementary Fig. 1f1/2).

Considering the RAS pathway, *PDGFC*, *KRAS*, *RAC2*, *SOS2*, and *PIK3R2* genes were upregulated in non-aggressive cases, only, whereas *RASA4*, *MET*, *PDGFA*, *RIN1*, *TIAM1*, *LAT*, and *BCL2L1* were upregulated in both groups (Supplementary Fig. 1g1/2).

Regarding the genes labeled as “driver genes,” there was an overlap for *ATRX*, *TRAF7*, *STK11*, *MET*, *SMAD2*, *CBL*, and *RUNX1*, with deregulation of several other genes in non-aggressive PTC, only (Supplementary Fig. 1h1/2). A similar picture was observed for the sub-analysis of the MAPK pathway, with upregulation of *TGFB1*, *PPP3CB*, *CACNB3*, *DUSP4*, *HSB1*, and *PDGFA* in both groups, but upregulation of *KRAS*, *MAP3K1*, *SOS2*, *DUSP5*, *RAC2*, and *CDC25B* and downregulation of *CACNA2D1* and *JUN* in the non-aggressive PTC group, only (Supplementary Fig. 1k1/2).

Finally, the PI3K pathway was significantly de-regulated in both groups, with 11 genes upregulated in both groups and several others de-regulated in non-aggressive cases (Supplementary Fig. 1l1/2).

## Discussion

In the present study, we reported that clinically aggressive PTC is associated with peculiar pathological features and gene expression profiles.

PTCs in general show indolent behavior with localized disease and typically do not recur or metastasize beyond locoregional lymph nodes. A recent meta-analysis, using univariate comparisons, has identified male sex, advanced age, tumor size, multifocality, vascular invasion, extra-thyroidal extension, and lymph node metastasis as risk factors for distant metastasis in well-differentiated thyroid cancers [24]. In our series, we could not confirm sex, age, and TNM stage as parameters of aggressiveness in the aggressive vs non-aggressive PTC group comparison, since these parameters were balanced in the case match procedure. Moreover, the relatively small sample size for each group—designed to be balanced for molecular analyses—and the not complete homogeneity of compositions of the two groups (i.e., RAI treatment) limit the statistical impact of clinical pathological correlates, as demonstrated by the low level of statistical significance in DFS and OS of non-aggressive and aggressive PTC cases. We could confirm multifocality and angioinvasion as parameters significantly more prevalent in aggressive PTC, the latter also showing a significant association with shorter disease-free survival.

Concerning additional pathological parameters, we found that the presence of TILs was more frequent in aggressive PTCs as compared to non-aggressive cases. This finding is in contrast with previous data on the association of immune cell infiltrate (including B and T cells) and a more favorable outcome [25–27]. As a limitation of our study, we did not perform subtyping of the tumor-associated immune compartment, but it is conceivable that our case series selection approach influenced our results as compared to literature studies. Moreover, our data are strengthened by the significant association of the presence of TILs with shorter disease-free survival (with a trend to significance also in terms of shorter overall survival).

Another feature associated with an aggressive clinical course in PTC was the presence of fibro-sclerotic tissue within the tumor. Our findings are supported by a few previous studies [28, 29] but are in contrast with a recent report on a large unselected series [30]. However, the value of our results is supported by a disease-free survival analysis that showed a significant adverse impact of the presence of extensive fibrotic changes. These fibrous tissue modifications were appearing as trabecular, net-like structures permeating tumor architecture. In a setting of parathyroid tumors, the presence of thick fibrous bands is commonly referred to as a sign of malignancy [31, 32].

Finally, increased mitotic activity and the presence of necrosis were identified as strong adverse prognostic indicators, both in disease-free and overall survival analyses, although their distribution was not statistically different comparing aggressive and non-aggressive PTC cases. This latter finding is strongly supportive of the concept of the high-grade differentiated thyroid cancer group, as a specific prognostic category within differentiated thyroid cancers and is in line with the novel category proposed by the fifth edition of the WHO book on endocrine tumors, which embeds aggressive differentiated carcinomas and poorly differentiated carcinomas in a single group named high-grade thyroid carcinomas [33, 34].

A second part of our study was focused on the definition of gene expression profiles in a subset of aggressive and non-aggressive PTC cases, selected from the pathological series and matched also for the histological subtype. Despite other studies employed NanoString nCounter technology to explore the molecular make-up of thyroid malignances [35–37], to the best of our knowledge, this is the first study using the nCounter^®^ PanCancer Pathways Panel to explore different canonical pathways in correlation with PTC aggressiveness. Our study falls in the scarce literature aimed at testing gene expression profiles as determinants of aggressiveness in PTC. The weakness of our approach is mostly based on the targeted approach (although covering over 700 genes), the lack of genomic data of the cases investigated, and the lack of RNA validation using an alternative approach or protein analysis by means of immunohistochemistry. By contrast, the strengths of our study are the robustness of the nanostring technology and the study design in terms of case selection. In fact, as compared to similar studies, we designed a comparative study on aggressive and non-aggressive PTC cases based on a strongly balanced and matched selection. A recent study based on TCGA data identified a specific signature of immune-related genes (namely *HSPA1A*, *NOX5*, and *FGF23*) associated with prognosis in PTC, but using data from a series of nearly 500 PTC cases with 3.2% of patients, only, died of disease and less than 2% of cases with distant metastases [38]. Another very recent study, also based on TCGA data, compared gene expression profiles in 455 non recurrent vs 46 recurrent PTC tumors specimens, identifying differential expression profiles in 40 genes with a significant influence of the tumor genotype [19]. In this study, recurrent PTC cases with BRAF-like signature significantly overexpressed—among other genes—*FN1*, *ITGA3*, and *MET* and downregulated TPO and TG.

Our study clearly shows that clinically aggressive and non-aggressive PTC cases in our series consistently share a gene expression signature as compared to normal thyroid tissue. In terms of pathway analysis, up-regulation of the RAS, MAPK, and PI3K pathways was consistent in both groups, although with an enrichment of de-regulated genes in the non-aggressive PTC group. However, a larger number of genes was deregulated in non-aggressive PTC as compared to normal tissues than those deregulated in aggressive PTC cases. The larger set of de-regulated genes depicted in non-aggressive PTC cases shows a more significant upregulation of genes belonging to the DNA damage repair and JAK/STAT pathways and a downregulation of genes belonging to the WNT pathway. Interestingly, the hedgehog pathway was differentially de-regulated in aggressive PTC as compared to non-aggressive PTC cases. In fact, whereas *WNT10A* and *GLI3* were significantly up- and down-regulated in aggressive PTC, *GSK3B* was upregulated in non-aggressive PTC cases.

These findings suggest that these genes are likely involved in molecular mechanisms of aggressiveness. In fact, WNT10A/beta-catenin pathway activation has been demonstrated to promote cellular characteristics of aggressiveness, such as proliferation and migration in thyroid cancer [39]. Data on GLI3-driven molecular mechanisms in thyroid cancer are not available. GLI3 activation/overexpression has been shown to promote tumor progression in colon cancer [40] but not in other models such as gallbladder or cervical cancer [41, 42] thus supporting tumor type-specific activities. On the other hand, *GSK3B* expression has been described to inhibit the progression of several cancer models, such as, among others, lung [43], gastric [44], and hepatocellular [45] cancers. Finally, *PKMYT1* was significantly upregulated in aggressive PTC cases, only. Although no data are available on the role of this gene in thyroid cancer, *PKMYT1* has been shown to exert an important effect on tumor immunity and progression in several cancer models [46].

In conclusion, we demonstrate that clinically aggressive PTC is characterized by peculiar pathological features, including an increased TILs infiltration and a more prevalent occurrence of intratumoral fibrosis, supporting the value of a careful reporting of these parameters in the PTC diagnostic work-up. Moreover, although non-aggressive PTC in our series showed a larger number of deregulated genes as compared to aggressive PTC cases, the existence of a set of differentially regulated genes in the two groups suggests that different molecular mechanisms are active in promoting clinical aggressiveness in PTC with special reference to the impairment of the hedgehog pathway. Although limited by the relative small sample size and biases in case selection, these results have a prime impact highlighting the need to evaluate the stromal component as part of the diagnostic workup for PTC, with special reference to the presence of fibrosclerotic changes, a pathological characteristic that is currently not considered in standardized pathology reporting for thyroid cancer [47]. Moreover, gene expression data pave the way for molecular validation studies targeting regulators of the hedgehog pathway to identify novel biomarkers of aggressiveness.

### Supplementary Information

Below is the link to the electronic supplementary material.

Supplementary file1 (PDF 1144 KB)

Supplementary file2 (DOCX 18 KB)

Supplementary file3 (DOCX 19 KB)

Supplementary file4 (XLSX 18 KB)

Supplementary file5 (XLSX 14 KB)

## Data Availability

Not applicable.
