# Supplementary material for: Clinical and Pathological Features and Gene Expression Profiles of Clinically Aggressive Papillary Thyroid Carcinomas
Source: Endocr Pathol. 2023 May 19;34(3):298–310. doi: 10.1007/s12022-023-09769-x (PMC10511602; doi:10.1007/s12022-023-09769-x)
Supplement: Supplementary file 1 — Supplementary file1 (PDF 1144 KB) [file 12022_2023_9769_MOESM1_ESM.pdf]

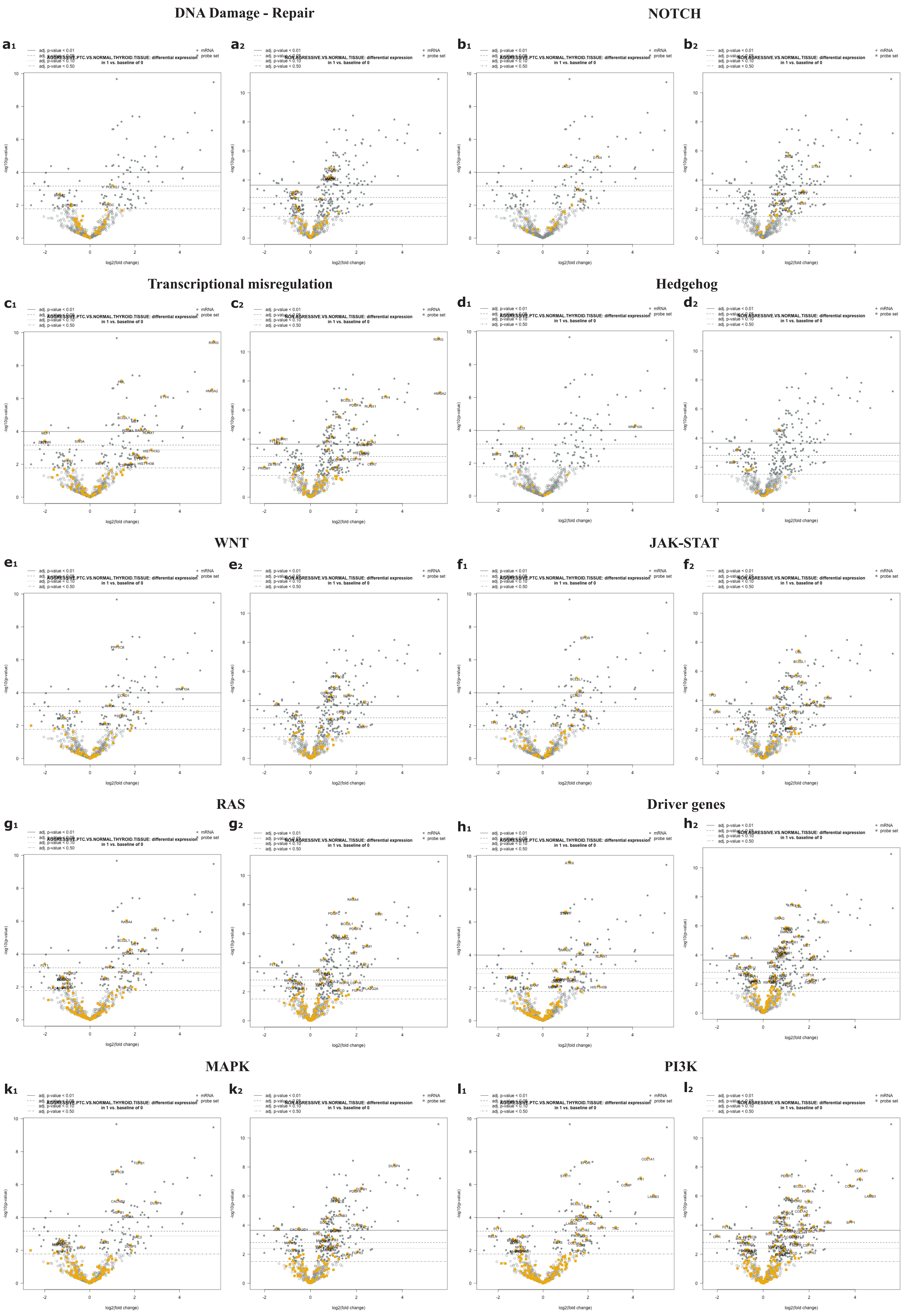

Supplementary Figure 1. Volcano plots illustrating differential expression of genes belonging to different pathways in aggressive PTC as compared to non-aggressive PTC cases.
