# Supplementary material for: Clinical and Pathological Features and Gene Expression Profiles of Clinically Aggressive Papillary Thyroid Carcinomas
Source: Endocr Pathol. 2023 May 19;34(3):298–310. doi: 10.1007/s12022-023-09769-x (PMC10511602; doi:10.1007/s12022-023-09769-x)
Supplement: Supplementary file 2 — Supplementary file2 (DOCX 18 KB) [file 12022_2023_9769_MOESM2_ESM.docx]

**Supplementary Table 1.** Characteristics of lymph node metastases.

|  |  | **Number of metastatic lymph nodes / total lymph nodes examined** | **Largest size of metastatic lymph node (mm)** | **Largest size of metastatic deposits (mm)** |
| --- | --- | --- | --- | --- |
| 1 | Non-aggressive PTC | 2/26 | 8 | 5 |
| 2 | Non-aggressive PTC | 2/13 | 13 | 12 |
| 3 | Non-aggressive PTC | 23/61 | 40 | 25 |
| 4 | Non-aggressive PTC | 1/4 | 6 | 5 |
| 5 | Non-aggressive PTC | 7/71 | 12 | 7 |
| 6 | Non-aggressive PTC | 3/7 | 5 | 2 |
| 7 | Non-aggressive PTC | 5/8 | 6 | 6 |
| 8 | Non-aggressive PTC | 1/6 | Not available | Not available |
| 9 | Non-aggressive PTC | 1/11 | 3 | 0.5 |
| 10 | Non-aggressive PTC | 5/6 | 10 | 10 |
| 11 | Non-aggressive PTC | 20/56 | 17 | 11 |
| 12 | Non-aggressive PTC | 2/13 | 6 | 5 |
| 13 | Non-aggressive PTC | 24/75 | 24 | 23 |
| 14 | Non-aggressive PTC | 4/8 | 7 | 6 |
| 15 | Non-aggressive PTC | 5/27 | 35 | 16 |
| 16 | Non-aggressive PTC | 2/6 | 6 | 2.5 |
| 17 | Non-aggressive PTC | 4/12 | 5 | 2 |
| 18 | Non-aggressive PTC | 1/19 | 11 | 1 |
| 19 | Non-aggressive PTC | 20/34 | 9 | 8 |
| 20 | Non-aggressive PTC | 11/18 | 9 | 0.6 |
| 21 | Non-aggressive PTC | 2/12 | 2 | 1 |
| 22 | Non-aggressive PTC | 3/8 | 3 | 2 |
| 23 | Non-aggressive PTC | 3/12 | Not available | Not available |
| 24 | Non-aggressive PTC | 2/7 | 19 | 19 |
| 25 | Non-aggressive PTC | 8/13 | Not available | Not available |
| 26 | Non-aggressive PTC | 7/15 | 20 | 12 |
|  |  |  |  |  |
| 1 | Aggressive PTC | 14/19 | 22 | 19 |
| 2 | Aggressive PTC | 3/3 | 25 | 13 |
| 3 | Aggressive PTC | 2/2 | 10 | 8 |
| 4 | Aggressive PTC | 3/3 | 25 | 10 |
| 5 | Aggressive PTC | 14/18 | 50 | 22 |
| 6 | Aggressive PTC | 1/2 | 10 | 6 |
| 7 | Aggressive PTC | 13/61 | 26 | 12 |
| 8 | Aggressive PTC | 1/5 | Not available | Not available |
| 9 | Aggressive PTC | 2/2 | 12 | 10 |
| 10 | Aggressive PTC | 1/4 | 11 | 1.5 |
| 11 | Aggressive PTC | 4/7 | 12 | 11 |
| 12 | Aggressive PTC | 2/3 | 15 | 10 |
| 13 | Aggressive PTC | 5/23 | 15 | 12 |
| 14 | Aggressive PTC | 6/32 | 26 | 20 |
| 15 | Aggressive PTC | 6/64 | 17 | 16 |
| 16 | Aggressive PTC | 1/13 | Not available | Not available |
| 17 | Aggressive PTC | 14/33 | 15 | 15 |
| 18 | Aggressive PTC | 5/49 | Not available | Not available |
| 19 | Aggressive PTC | 7/15 | Not available | Not available |
| 20 | Aggressive PTC | 4/5 | 12 | 12 |
| 21 | Aggressive PTC | 7/24 | 19 | 18 |
| 22 | Aggressive PTC | 8/22 | 20 | 11 |
| 23 | Aggressive PTC | 6/6 | 21 | 20 |
| 24 | Aggressive PTC | 23/62 | 29 | 29 |
| 25 | Aggressive PTC | 18/23 | 15 | 14 |
| 26 | Aggressive PTC | 10/34 | 35 | 22 |
