# Supplementary material for: Clinical and Pathological Features and Gene Expression Profiles of Clinically Aggressive Papillary Thyroid Carcinomas
Source: Endocr Pathol. 2023 May 19;34(3):298–310. doi: 10.1007/s12022-023-09769-x (PMC10511602; doi:10.1007/s12022-023-09769-x)
Supplement: Supplementary file 3 — Supplementary file3 (DOCX 19 KB) [file 12022_2023_9769_MOESM3_ESM.docx]

**Supplementary Table 2.** Clinical and pathological characteristics of cases included in nCounter® Nanostring analyses (#48).

| Parameters | | TOTAL | Non-aggressive PTC | Aggressive PTC | P value |
| --- | --- | --- | --- | --- | --- |
| Age median (interval) | | 38 (18-73) | 37 (18-61) | 40 (19-73) | / |
| Sex | M | 16 | 8 | 8 | / |
|  | F | 32 | 16 | 16 |  |
| Variant | Classic / Follicular | 38 | 19 | 19 | / |
|  | Other histotypes | 10 | 5 | 5 |  |
| T | 1 | 30 | 15 | 15 | / |
|  | 2 | 16 | 8 | 8 |  |
|  | 3 | 2 | 1 | 1 |  |
| N | 0 | 22 | 11 | 11 | / |
|  | 1 | 26 | 13 | 13 |  |
| Tumor diameter  (cm) | ≤1 | 10 | 6 | 4 | 0.702 |
|  | > 1 - ≤ 2 | 22 | 9 | 13 |  |
|  | > 2 - ≤ 4 | 14 | 8 | 6 |  |
|  | > 4 | 2 | 1 | 1 |  |
| Multifocal presentation | no | 24 | 19 | 5 | <0.001 |
|  | yes | 24 | 5 | 19 |  |
| Bilateral presentation | no | 33 | 20 | 13 | 0.029 |
|  | yes | 15 | 4 | 11 |  |
| Tumor capsule | absent | 22 | 7 | 15 | 0.064 |
|  | incomplete | 19 | 12 | 7 |  |
|  | complete | 7 | 5 | 2 |  |
| Extrathyroidal extension | no | 23 | 14 | 9 | 0.149 |
|  | yes | 25 | 10 | 15 |  |
| Vascular invasion | no | 22 | 14 | 8 | 0.082 |
|  | yes | 26 | 10 | 16 |  |
| Surgical margins | negative | 35 | 18 | 17 | 0.745 |
|  | positive | 13 | 6 | 7 |  |
| Necrosis | no | 47 | 23 | 24 | 0.312 |
|  | yes | 1 | 1 | 0 |  |
| Mitosis | 0 | 8 | 6 | 2 | 0.199 |
|  | 1 | 39 | 18 | 21 |  |
|  | 2 | 1 | 0 | 1 |  |
| Tumor infiltrating lymphocytes | no | 24 | 17 | 7 | 0.004 |
|  | yes | 24 | 7 | 17 |  |
| Sclerosis | absent | 16 | 10 | 6 | 0.040 |
|  | moderate | 18 | 11 | 7 |  |
|  | extensive | 14 | 3 | 11 |  |
| Follow-up | alive | 45 | 23 | 22 | 0.551 |
|  | dead | 3 | 1 | 2 |  |
